# Comparison of Intravenous Ibuprofen Versus Intravenous Ketorolac in Acute Postoperative Pain: A Systematic Review and Meta-Analysis of Randomized Controlled Trials

**DOI:** 10.7759/cureus.73759

**Published:** 2024-11-15

**Authors:** Abhijit Nair, Abhay Bodhey, Ahmed A Jabri, Faisal Al Sawafi, Ujjwalraj I Dudhedia

**Affiliations:** 1 Anesthesiology, Ibra Hospital, Ibra, OMN; 2 Anesthesiology, Rashid Hospital, Dubai, ARE; 3 Emergency Medicine, Ibra Hospital, Ibra, OMN; 4 Critical Care, Ibra Hospital, Ibra, OMN; 5 Anesthesiology, Dr. L. H. Hiranandani Hospital, Mumbai, IND

**Keywords:** acute postoperative pain, ibuprofen, ketorolac tromethamine, (nsaid) non-steroidal anti-inflammatory drugs, systematic review and meta analysis

## Abstract

Non-steroidal anti-inflammatory drugs (NSAIDs) are popularly used in the management of acute postoperative pain. Intravenous (IV) ketorolac has been used for several years for this purpose. Recently, IV ibuprofen has been introduced for the management of postoperative pain. This review aims to compare the efficacy of these two NSAIDs in managing acute postoperative pain. After registering the protocol in the International Prospective Register of Systematic Reviews (PROSPERO), databases like PubMed, the Cochrane Central Register of Controlled Trials (CENTRAL), the Cumulative Index to Nursing and Allied Health Literature (CINAHL), and Ovid were searched using relevant keywords. Twenty-four-hour opioid consumption was the primary outcome. Pain scores, patient satisfaction, rescue analgesia requirements, and adverse events were the secondary outcomes assessed. Out of 124 articles that were retrieved, six articles fulfilled the inclusion criteria. The Risk of Bias 2 (RoB-2) was used for risk of bias assessment, Review Manager (RevMan) was used for a quantitative meta-analysis, and the Grading of Recommendations Assessment, Development, and Evaluation (GRADE) was used to assess the strength of evidence. The risk of bias was high in all categories. The 24-hour opioid requirement, which was the primary outcome, was comparable between both groups (mean difference: -4.72; 95% CI: -5.65, -3.80; P=0.79), with significant heterogeneity (I^2^=93%). The secondary outcomes were comparable among both groups. The Grading of Recommendations, Assessment, Development, and Evaluations (GRADE) strength of evidence was moderate for the pain score at movement and low to very low for other outcomes. Based on the results of this review, the efficacy of IV ibuprofen and IV ketorolac are comparable. However, the findings should be interpreted with caution due to significant clinical and statistical heterogeneity. Well-designed, adequately powered studies need to be conducted to find out the dose, frequency, and type of surgery suitable for various NSAIDs.

## Introduction and background

Acute postoperative pain is a significant concern in surgical recovery, influencing patient comfort, rehabilitation, and overall outcomes. Nonsteroidal anti-inflammatory drugs (NSAIDs) play a crucial role in multimodal analgesia strategies, helping to reduce the need for opioids, minimize side effects, and improve pain control [1]. Ibuprofen and ketorolac are both NSAIDs that work by inhibiting the cyclooxygenase (COX) enzymes, specifically COX-1 and COX-2, to produce their analgesic and anti-inflammatory effects. Their affinities for COX enzymes differ, though. Ibuprofen has a more balanced COX-1/COX-2 inhibition profile, whereas ketorolac is known to be a more potent COX-1 inhibitor. Variations in enzyme inhibition may lead to variations in side effects and efficacy [2,3]. Several studies have compared the efficacy of intravenous (IV) ketorolac and IV ibuprofen in managing postoperative pain across various surgical settings. Ketorolac is considered one of the most potent NSAIDs for short-term pain relief. It has been shown to provide significant analgesia, often comparable to opioids, particularly in the early postoperative period [4-6].

IV ibuprofen, on the other hand, is a newer formulation that has demonstrated efficacy in reducing pain and opioid consumption [7-9]. A key advantage of ibuprofen is its potential for sustained analgesia with fewer gastrointestinal and bleeding risks compared to ketorolac, especially in patients requiring longer NSAID administration [10]. Systematic reviews and meta-analyses have found that both drugs are effective in managing moderate to severe acute postoperative pain [11,12].

This review aims to compare the analgesic efficacy of IV ibuprofen with that of IV ketorolac when used for managing acute postoperative pain in adult patients.

## Review

Methods

The methodological framework for this systematic review and meta-analysis was designed following the Preferred Reporting Items for Systematic Review and Meta-Analysis (PRISMA) guidelines [13]. On September 14, 2024, the study protocol (registration number: CRD42024586638) was registered with the International Prospective Register of Systematic Reviews (PROSPERO) network. This study was carried out following the PRISMA statement guidelines and the protocol suggested by the Cochrane Collaboration.

The primary outcome of this review was to compare the analgesic efficacy of IV ibuprofen with IV ketorolac in adult patients undergoing elective surgeries in terms of 24-hour opioid consumption. The secondary outcomes included pain scores at various time frames, patient satisfaction scores, length of hospital stay, and adverse events like postoperative nausea/vomiting (PONV), bleeding, gastrointestinal symptoms, and renal impairment.

The inclusion and exclusion criteria were determined before commencing the study, with only randomized controlled trials (RCTs) that compared IV ibuprofen with IV ketorolac eligible for inclusion. The PICO criteria of this study are as follows: Patients (P) include adult patients undergoing elective surgery; Intervention (I) involves IV ibuprofen for perioperative analgesia; Comparison (C) comprises IV ketorolac for postoperative analgesia; Outcome measurements (O) cover postoperative pain scores, 24-hour opioid consumption, time to rescue analgesia, and adverse events; and the study design (S) consists of RCTs.

Studies were excluded if they 1) included pediatric patients; 2) compared IV ibuprofen with a placebo or any other analgesic; 3) were case reports, case series, editorials, letters to the editor, reviews, or animal or laboratory studies. Relevant keyword searches for RCTs from January 2000 to August 2024 were conducted in PubMed, CINAHL, Ovid, and the Cochrane Library (CENTRAL). Table 1 presents a comprehensive search strategy.

**Table 1:** Details of all database searches.

Database	Search details
PubMed	((ibuprofen) AND (ketorolac)) AND (intravenous) Filters: from 2000/1/1 - 2024/9/30 Sort by: Most Recent (("ibuprofen"[MeSH Terms] OR "ibuprofen"[All Fields] OR "ibuprofen s"[All Fields] OR "ibuprofens"[All Fields]) AND ("ketorolac"[MeSH Terms] OR "ketorolac"[All Fields]) AND ("intraveneous"[All Fields] OR "intraveneously"[All Fields] OR "intravenous"[All Fields] OR "intravenously"[All Fields])) AND (2000/1/1:2024/9/30[pdat]) Translations ibuprofen: "ibuprofen"[MeSH Terms] OR "ibuprofen"[All Fields] OR "ibuprofen's"[All Fields] OR "ibuprofens"[All Fields] ketorolac: "ketorolac"[MeSH Terms] OR "ketorolac"[All Fields] intravenous: "intraveneous"[All Fields] OR "intraveneously"[All Fields] OR "intravenous"[All Fields] OR "intravenously"[All Fields]
OViD	ibuprofen AND ketorolac AND acute pain {No related terms}
CINAHL	ibuprofen AND ketorolac AND acute pain Limiters: Publication Date: 20000101-20240831
Cochrane Library	"ibuprofen" in Title Abstract Keyword AND "ketorolac tromethamine" in Title Abstract Keyword AND "postoperative" in Title Abstract Keyword (Word variations have been searched) "ibuprofen":ti,ab,kw AND "ketorolac tromethamine":ti,ab,kw AND "postoperative":ti,ab,kw (Word variations have been searched)

Data Extraction

The titles and abstracts of the reports that were found were independently scanned by two investigators (AN and AB). The complete text of a report was obtained and assessed if it was deemed eligible based on its title or abstract. Any abstract that did not provide enough details about the eligibility requirements was selected for full-text review. At least one researcher found potentially relevant studies, which were then retrieved, and their full-text versions assessed. The two investigators (AN and AB) independently evaluated each article that satisfied the inclusion criteria, and disagreements were settled by discussion. A third investigator (AJ) resolved disagreements regarding inclusion or exclusion.

Using a standardized data collection form, two independent investigators (AN and AB) extracted all relevant data from the included studies, entered them into standardized forms, and then cross-checked them. Any discrepancy was resolved through discussion. In case of disagreement, a third investigator (FAS) provided a decision. If included studies used opioids other than morphine as rescue analgesic, they were converted to IV morphine equivalents using an online calculator (https://clincalc.com/Opioids/).

Risk of Bias Assessment

Two independent authors (AN and AB) used the Revised Cochrane Risk of Bias (RoB) tool for randomized trials to evaluate the risk of bias [14]. Bias resulting from the randomization process (D1), deviations from the intended interventions (D2), missing outcome data (D3), discrimination in outcome measurement (D4), and bias in the selection of the reported result (D5) comprise the five domains that constitute the risk of bias. When the risk of bias for all domains was low, the risk was considered low risk; when the risk of bias for at least one domain was high, the risk of biases for multiple domains was considered some concern; and if the overall judgment was neither low nor high, it was considered some concern.

Strength of Quality Across All Trials

The Grading of Recommendation, Assessment, Development, and Evaluation (GRADE) guidelines were used to evaluate the overall methodological quality of the evidence across pooled outcomes [15]. The evidence for pooled outcomes was determined by taking into account the study design, bias risk, consistency, directness, precision, and other factors namely confounding, large effect, and publication bias). The degree of certainty in the evidence was defined as follows: (1) additional research of high quality is unlikely to change the estimate of the effect's confidence; (2) additional research of moderate quality is likely to have a significant impact on the estimate's confidence and may change it; (3) additional research of low quality is likely to change the estimate; or (4) extremely poor quality: the estimate is uncertain.

Quantitative Meta-analysis

After a qualitative review, a quantitative review was conducted for all included studies that directly compared IV ibuprofen with IV ketorolac.

Statistical Analysis

The Mantel-Haenszel technique was used to assess dichotomous variables, and the risk ratio with the associated 95% confidence interval (CI) was determined. For units-unified continuous variables, the mean difference (MD) with the accompanying 95% CI was determined using the inverse variance approach. When P > 0.01 and I^2 < 50%, the fixed effects model was used for meta-analysis. We evaluated the heterogeneity between studies using the I^2 statistic, which was defined as: 0-40% might not be important, 30-60% may represent moderate heterogeneity, 50-90% may represent significant heterogeneity, and 75-100% considerable heterogeneity [16]. A P-value of less than 0.05 was considered statistically significant for all statistical tests. Review Manager version 5.4.1 (Cochrane Collaboration, Software Update, Oxford, UK) was used for analysis [17].

Sensitivity Analysis and Publication Bias

The reliability of the combined results was subsequently evaluated based on the degree of consistency of the results after they were compared with the random effects and fixed effects models. The fixed effects model was applied when P>0.01 and I2<50%, and the random effects model was applied for meta-analysis when P<0.01 and I^2^>50%. To determine if there was publication bias, a funnel plot will be generated if there are more than ten studies that fulfill the inclusion criteria.

Results

**Figure 1:**
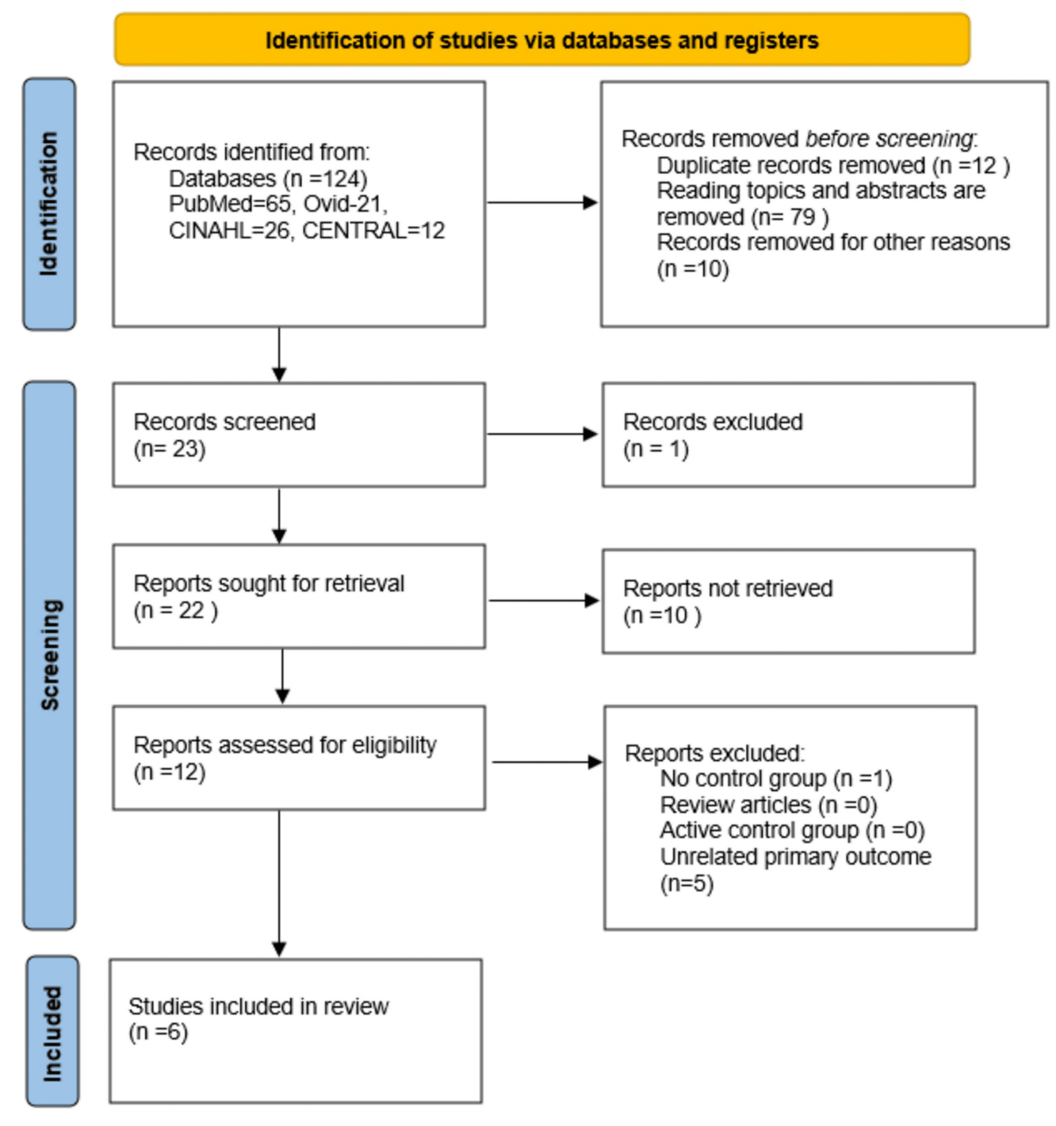
PRISMA flowchart. PRISMA: Preferred Reporting Items for Systematic Review and Meta-Analysis.

Using the previously stated inclusion criteria, 124 articles were identified. Figure 1 shows the PRISMA flow diagram of the entire database search and the final selection of articles. One title was eliminated from the 23 titles that were screened after duplicates and irrelevant articles were eliminated. Ten of the 22 remaining articles could not be retrieved because they were deemed irrelevant. Of the remaining articles, six articles were excluded (one with no control group, and five with unrelated primary outcomes). Finally, six articles (RCTs) were selected for a qualitative systematic review and a quantitative meta-analysis [18-23]. Study characteristics and outcome details are summarized in Tables 2-3.

**Table 2:** Summary of all the included studies. NSAIDs: Non-steroidal anti-inflammatory drugs; GA: General anesthesia; PCA: Patient-controlled analgesia; VAS: Visual analog scale.

Author(s) and year	Country	Study period	Number of patients	Intervention (IV Ibuprofen), dose/frequency	Control (IV ketorolac)	Surgeries performed	Anaesthesia for surgery	Reported outcomes
Dwarica DS et al. [18](2020)	United States	September 2013- May 2015	224	112 patients, 800 mg 8^th^ hourly	112 patients, 30 mg 8^th^ hourly	Urogynaecology surgeries	GA	Primary outcome: pain and satisfaction with pain control (VAS). Secondary outcomes: opioid consumption, length of stay
Ali A et al. [19] (2022)	Pakistan	January 2021- June 2021	68	34 patients, 800 mg 8^th^ hourly	34 patients, 30 mg 8^th^ hourly	Abdominal surgeries	GA	Primary outcome: postoperative pain scores. Other outcomes- analgesic consumption.
Amin C et al. [20](2024)	Egypt	November 2022- May 2023	96	46 patients, 800 mg 6^th^ hourly	50 patients, 30 mg 6^th^ hourly	Open elective abdominal hysterectomy with or without salpingo-oophorectomy	GA	Primary outcome: mean postoperative dynamic VAS during the first 24 hours. Secondary outcomes:static and dynamic VAS, time to first analgesic requirement intraoperative fentanyl requirements, postoperative morphine requirements, time to independent movement.
Uribe AA et al. [21](2018)	United States	September 2012- December 2012	51	20 patients, 800 mg 2 doses	31patients, 30 mg single dose	Arthroscopic knee surgery	GA	Primary outcome: to compare the analgesic efficacy of two NSAIDs. Secondary outcomes: postoperative opioid consumption, patients receiving rescue analgesia.
Veronica MD et al. [22] (2016)	United States	--	48	25 patients, 800 mg 6^th^ hourly	23 patients, 30 mg 6^th^ hourly	Cesarean section	Spinal anaesthesia	Primary outcome: need for rescue analgesia) in terms of PCA attempts). Secondary outcomes: postoperative analgesic consumption.
Shehab AS et al. [23] (2024)	Egypt	December 2022- March 2023	50	25 patients, 800 mg 6^th^ hourly	25 patients, 30 mg 6^th^ hourly	Cesarean section	GA	Primary outcome: to evaluate the effectiveness of both NSAIDs. Secondary outcomes: 24-h opioid requirements, time to ambulate, and the time to first rescue analgesic dose.

**Table 3:** Summary of all the outcomes in the included studies. I: Ibuprofen; K: Ketorolac; PONV: Postoperative nausea/vomiting; LOS: Length of stay.

S.no.	Study	Groups	No. of patients	Opioid consumption	Pain score (rest) 1^st^ day	Pain score (movement) 1^st^ day	Pain score in the recovery room	No. of patients needing morphine	Patient Satisfaction	Time to rescue analgesia	PONV	LOS
1	Dwarica DS et al. [18](2020)	I	112	12 +/- 15	2.68 +/- 2.34	4.16 +/- 2.73	--	--	4.67 +/- 2.57	--	--	30.29 +/- 11.26
		K	112	11 +/- 14	2.30 +/- 2.1	3.94 +/- 2.57	--	--	2.77 +/- 2.42	--	--	32.98 +/- 15.11
2	Ali A et al. [19](2022)	I	34	--	--	3.00 +/- 0.82	--	--	--	--	--	--
		K	34	--	--	3.30 +/- 0.67	--	--	--	--	--	--
3	Amin S et al. [20] (2024)	I	46	6 +/- 4.8	0.9 +/- 0.52	1.1 +/- 0.74	--	46/46	5 +/- 2.23	--	10/46	--
		K	50	6 +/- 4.7	0.7 +/- 0.52	1+/- 0.44	--	49/50	6 +/- 2.23	--	8/50	--
4	Uribe AA et al. [21] (2018)	I	20	12.41+/- 16.56	--	--	0.9 +/- 1.71	11/20	--	77.62 +/- 33.03	--	--
		K	31	11.25 +/- 15.91	--	--	3.3 +/- 2.98	26/31	--	55.78 +/- 35.37	--	--
5	Veronica MD et al. [22] (2016)	I	25	21 +/- 28	--	--	--	--	--	--	--	--
		K	23	13 +/- 15	--	--	--	--	--	--	--	--
6	Shehab AS et al. [23] (2024)	I	25	4 +/- 2	4.32 +/- 1.3	4.56 +/- 1.92	--	--	--	328.0 +/- 89.63	--	--
		K	25	11 +/- 2	3.17 +/- 1.08	3.21 +/- 1.11	--	--	--	158.40 +/- 57.13	--	--

Risk of Bias Assessment

**Figure 2:**
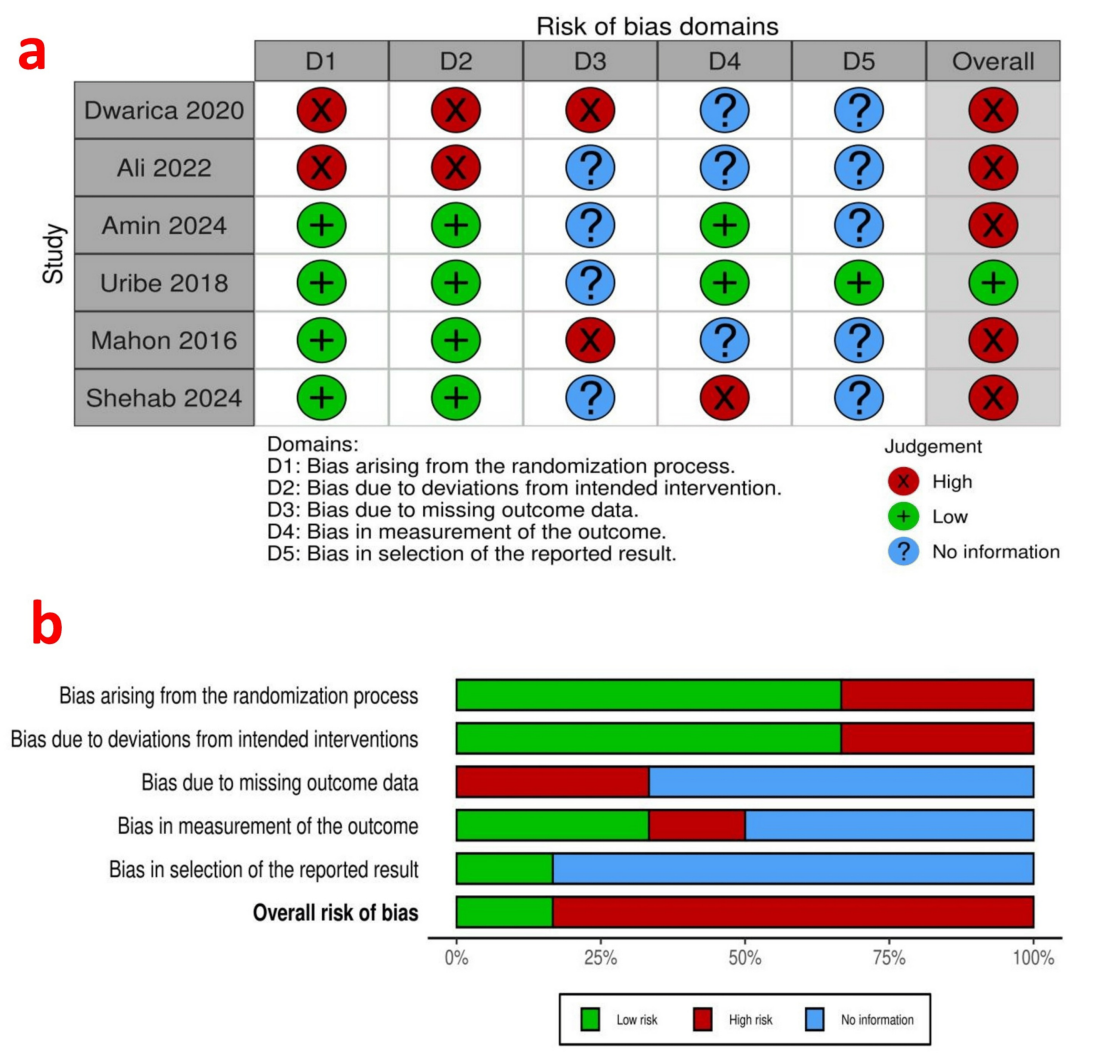
Risk of bias details. a: Traffic-light plot [18-23], b: Summary plot.

Figure 2 depicts the traffic-light plot (2a) and the summary plot (2b). The bias from the randomization process was low in four studies [20-23] and high in two studies [18,19]. Bias due to deviations from intended interventions (allocation concealment) was low in four studies [20-23] and high in two studies [18,19]. Bias arising from missing outcome data was low in four studies [19,21,22,24], high in two studies [18,20], and there was no information in four studies [19-21,23]. Bias in the outcome measurement was low in two studies [20,21], high in one study [23], and there was no information in three studies [18,19,22]. Bias arising from the selection of reported results was low in one study [21] and no information in five studies [18-20,22].

Quality of Evidence

GRADE assessment was performed for five outcomes: 24-hour opioid consumption, overall pain score at rest and movement, patients requiring postoperative opioids, and patient satisfaction score (Table 4). The level of evidence was moderate for pain score at movement, low for 24-hour opioid consumption, and very low for the remaining three outcomes.

**Table 4:** GRADE level of evidence. Strength of evidence: ⨁⨁◯◯- Low, ⨁◯◯◯- Very low, ⨁⨁⨁◯- Moderate a. There is a high risk of bias in random sequence generation, allocation concealment, and outcome data assessment. b. The surgeries and anesthesia management were heterogeneous. CI: Confidence interval; MD: Mean difference.

Certainty assessment							No. of patients		Effect		Certainty	Importance
No. of studies	Study design	Risk of bias	Inconsistency	Indirectness	Imprecision	Other considerations	IV ibuprofen	IV ketorolac	Relative (95% CI)	Absolute (95% CI)		
24-hour opioid consumption												
5	Randomised trials	Serious^a^	Seriousb	Serious^a^	Seriousb	Strong association all plausible residual confounding would reduce the demonstrated effect	228	2411	-	MD 4.72 lower (5.65 lower to 3.8 lower)	⨁⨁◯◯ Low^a,b^	CRITICAL
Pain scores at rest												
3	Randomised trials	Serious^a^	Serious^a^	Serious^b^	Serious^b^	All plausible residual confounding would reduce the demonstrated effect	183	187	-	MD 0.3 higher (0.11 higher to 0.48 higher)	⨁◯◯◯ Very low^a,b^	CRITICAL
Pain scores at movement												
4	Randomised trials	Serious^a^	Serious^b^	Serious^b^	Serious^a^	Strong association all plausible residual confounding would reduce the demonstrated effect dose response gradient	217	221	-	MD 0.05 higher (0.13 lower to 0.24 higher)	⨁⨁⨁◯ Moderate^a,b^	CRITICAL
Patients requiring postoperative opioids												
2	Randomised trials	Serious^a^	Serious^a,b^	Serious^a,b^	Serious^b^	All plausible residual confounding would reduce the demonstrated effect	57/66 (86.4%)	75/81 (92.6%)	not estimable		⨁◯◯◯ Very low^a,b^	CRITICAL
								0.00%				
Patient satisfaction score												
2	Randomised trials	Serious^a,b^	Serious^a^	Serious^a,b^	Serious^b^	All plausible residual confounding would reduce the demonstrated effect	158	162	-	MD 0.47 higher (2.38 lower to 3.31 higher)	⨁◯◯◯ Very low^a,b^	CRITICAL

Quantitative Meta-Analysis

A total of six articles fulfilled inclusion criteria and the data available was subjected to a quantitative meta-analysis (314 patients in the ibuprofen group and 336 patients in the ketorolac group) [18-23].

Primary Outcome Meta-Analysis

**Figure 3:**
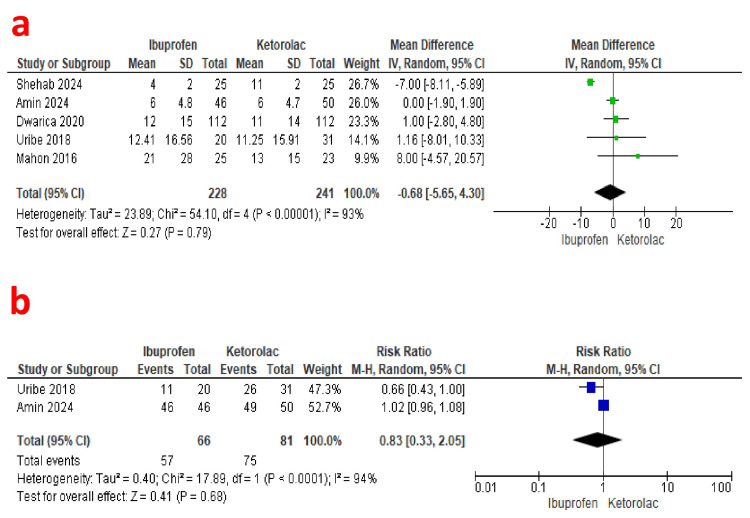
Forest plot showing the comparison of 24-hour opioid consumption and postoperative opioid requirement between the ibuprofen and ketorolac groups. a [18,20-23]: Comparison of 24-hour opioid consumption between the ibuprofen and ketorolac groups. b [20,21]: Comparison of postoperative opioid requirement between the ibuprofen and ketorolac groups.

24 hours opioid consumption: 24-hour opioid consumption was the primary outcome of this study. Five studies reported 24-hour opioid consumption as an outcome (228 patients in the ibuprofen group and 241 patients in the ketorolac group) [18, 20-23]. A pooled analysis depicted comparable opioid consumption over 24 hours in both groups (MD: -4.72; 95% CI: -5.65, -3.80; P=0.79). A random effects model revealed significant heterogeneity (I2=93%) (GRADE: low) (Figure 3a).

Opioid requirement postoperatively: The postoperative opioid requirement was reported by two studies (57 patients in the ibuprofen group and 75 patients in the ketorolac group) [20,21]. A pooled analysis revealed comparable opioid requirements in both groups (Risk Ratio: 0.83; 95% CI: 0.33, 2.05; P=0.68). A random effects model revealed significant heterogeneity (I2=94%) (GRADE: very low) (Figure 3b).

**Figure 4:**
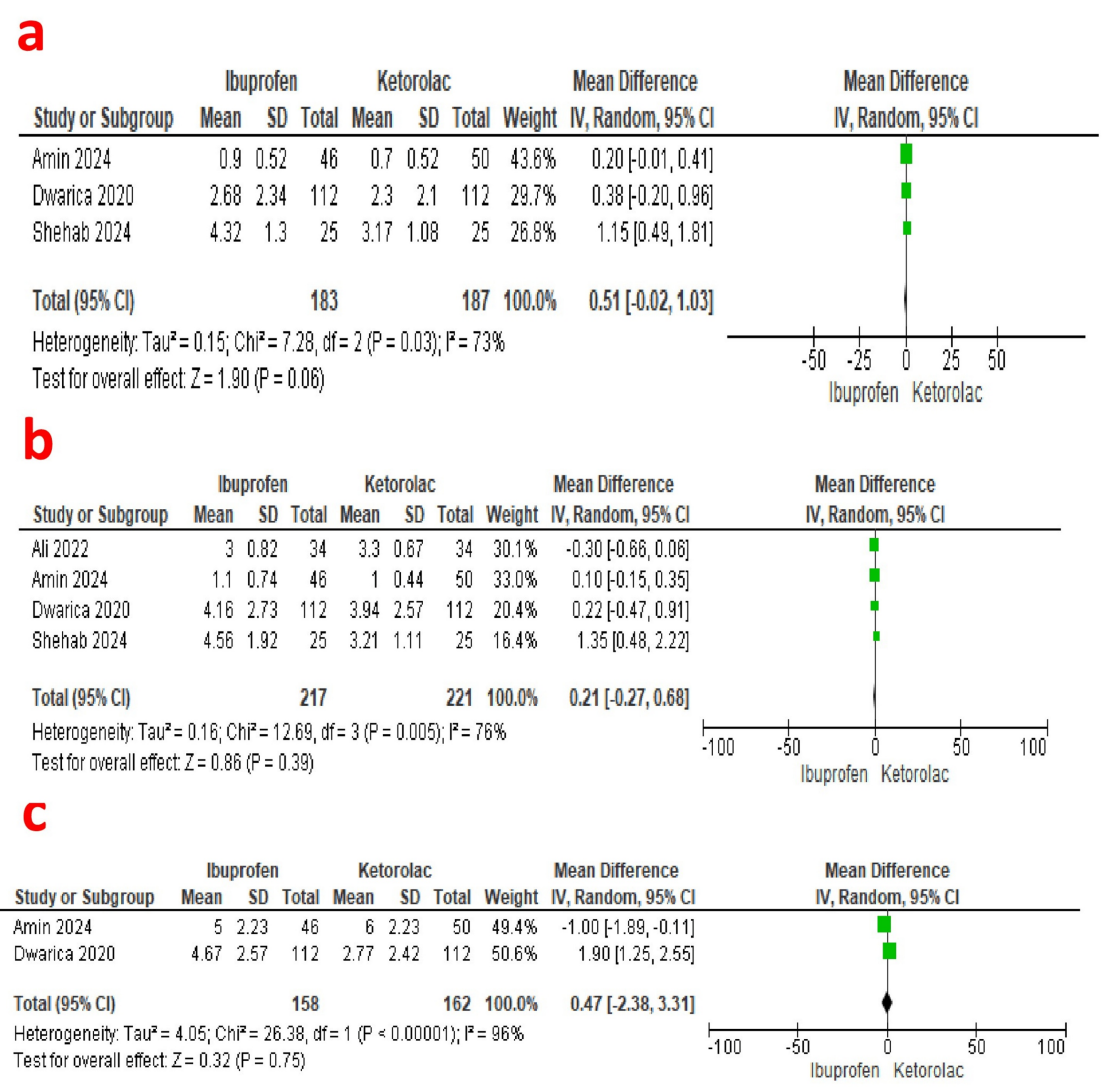
Forest plot showing the comparison of overall pain score at rest and at movement, and patient satisfaction scores between the ibuprofen and ketorolac groups. a [18,20,23]: Comparison of overall pain score at rest between the ibuprofen and ketorolac groups. b [18-20,23]: Comparison of overall pain score at movement between the ibuprofen and ketorolac groups. c [18,20]: Comparison of patient satisfaction scores between the ibuprofen and ketorolac groups.

Pain score at rest on the first day: Pain score at rest on the first day was reported by three studies (183 patients in the ibuprofen group and 187 patients in the ketorolac group) [18,20,23]. A pooled analysis revealed comparable pain scores in both groups (Mean Difference: 0.51; 95% Confidence Interval: 0.02, 1.03; P=0.06). A fixed-effect model revealed no heterogeneity (I2=73%) [GRADE: very low] (Figure 4a).

Pain score at movement on the first day: Four studies reported pain scores at movement on the first day (217 patients in the ibuprofen group and 221 patients in the ketorolac group) [18-20,23]. A pooled analysis revealed comparable pain scores in either group (Mean Difference: 0.21; 95% Confidence Interval: -0.27, 0.68; P=0.39). A fixed-effect model revealed significant heterogeneity (I2=76%) [GRADE: moderate] (Figure 4b).

Patient satisfaction: Patient satisfaction was reported by two studies (158 patients in the ibuprofen group and 162 patients in the ketorolac group) [18,20]. A pooled analysis revealed comparable patient satisfaction scores in either group (Mean Difference: 0.47; 95% Confidence Interval: -2.38, 3.31; P=0.75). A fixed-effect model revealed significant heterogeneity (I2=96%) [GRADE: very low] (Figure 4c).

Sensitivity Analysis and Publication Bias

To ensure that the pooled effect sizes were not the result of a single predominating study, data from each study were sequentially removed. The robustness of the pooled estimates for outcomes that included data from three or more studies was then assessed by reanalyzing the remaining data. There was no statistically significant difference. Publication bias assessment was not conducted as the number of eligible studies was less than 10.

Discussion

This systematic review and meta-analysis compared the analgesic efficacy of IV ibuprofen with IV ketorolac when used as an analgesic for various surgeries. A pooled analysis revealed that 24-hour opioid consumption was comparable in both groups, which was the primary outcome. The other outcomes were also comparable between both groups. The strength of this review was that only RCTs were utilized for analysis. The dose and the frequency of the NSAIDs were consistent in all the studies. However, the overall risk of bias was very high, and the strength of evidence was moderate to very low. There was significant statistical and clinical heterogeneity among the included patients in various studies.

As part of a multimodal analgesia strategy, NSAIDs are frequently used in postoperative pain management. They have an opioid-sparing effect, which reduces the negative effects of opioids and provides significant pain relief following major surgery. NSAIDs are generally well tolerated, but their impact on thrombotic events, perioperative bleeding, and the risk of acute kidney injury (AKI) are concerns [24,25]. Ketorolac was the only NSAID approved for IV treatment of postoperative pain in the US and many other countries until 2009. Ketorolac can be administered orally or parenterally as an intramuscular (IM) injection or IV. Although it has only mild anti-inflammatory effects, it is a strong analgesic with a short half-life of four to six hours and a quick onset (30 to 60 minutes) [26].

Diclofenac is another NSAID that is extensively used and can be administered orally, IV/IM, and per-rectally. Alexander R et al. compared the total amount of morphine consumed, pain scores, and opioid side effects in 102 total knee and total hip arthroplasty patients who received IV diclofenac, IV ketorolac, or a placebo before surgery in a double-blind, randomized control trial [27]. They demonstrated that patients taking diclofenac and ketorolac had significantly fewer opioid side effects and consumed significantly less morphine than the placebo group. Forrest JB et al. compared the serious adverse effects of ketorolac with diclofenac or ketoprofen in adult patients undergoing elective major surgeries [28]. The study included 11,245 patients from 49 European hospitals. The authors concluded that the safety of ketorolac was comparable to that of ketoprofen and diclofenac for the treatment of pain after major surgery.

The U.S. FDA approved intravenous ibuprofen in 2009 to treat mild to moderate pain, moderate to severe pain when combined with opioid therapy, and to reduce fever in adults [29]. In 2015, pediatric patients aged 6 months and older were added to the list of indications [30]. In a narrative review by Southworth and Sellers, the authors summarized the findings of nine clinical trials comprising 1062 patients in whom IV ibuprofen was compared with either a placebo or a comparable medication [31]. The authors concluded that IV ibuprofen should be considered in the analgesic regimen for the management of postoperative pain due to its favorable safety profile, which includes fewer serious adverse events, significantly lower levels of perioperative cytokines and catecholamines release, and its opioid-sparing properties. A total of 406 patients scheduled for elective, single-site orthopedic or abdominal surgeries participated in a multicenter, randomized, double-blind, placebo-controlled study by Southworth S et al. [32]. Patients were randomly assigned to receive either 400 mg of IV ibuprofen every six hours (for a total of 8 doses in 48 hours), 800 mg of IV ibuprofen every six hours (for a total of 8 doses in 48 hours), or a placebo. Ibuprofen 800 mg IV every six hours was found to significantly reduce pain at rest and with movement in three time periods (1-24, 6-24, and 12-24 hours) when compared to a placebo. In contrast, ibuprofen 400 mg IV every six hours significantly reduced pain at rest and with movement in the 6- to 24-hour and 12- to 24-hour periods.

In a retrospective study of patients undergoing laparoscopic cholecystectomy, Lee GG et al. compared findings from 77 patients who received 400 mg of IV ibuprofen and 86 patients who received 30 mg of IV ketorolac for postoperative analgesia [33]. The analysis of the retrospective data led the authors to conclude that the postoperative pain score in the recovery room was significantly higher in the ibuprofen group than in the ketorolac group (p = 0.027). Additionally, the number of patients who required immediate rescue analgesics in the recovery room was higher in the ibuprofen group than in the ketorolac group (p = 0.036). A possible reason for these findings could be the dose of ibuprofen used, which was 400 mg, half of what was used in most prospective studies. Zhou HS et al. compared the safety and efficacy of 400 mg and 800 mg of IV ibuprofen with a placebo in patients undergoing abdominal and orthopedic surgeries with incisions of at least 5 cm, who were hospitalized after the surgery [34]. The authors concluded that administering 400 mg and 800 mg of IV ibuprofen perioperatively every six hours (a total of 8 doses) reduced postoperative morphine consumption and provided effective pain relief. Adverse events such as gastrointestinal symptoms, bleeding, nephrotoxicity, and other cardiovascular events were comparable between both doses and the placebo.

Zhou P et al. published a systematic review and meta-analysis investigating the safety and efficacy of IV ibuprofen for managing postoperative pain and fever [35]. The review analyzed data from 23 RCTs involving a total of 3716 patients. Based on the results, the authors concluded that there is moderate-to-low certainty evidence supporting the use of IV ibuprofen in adults with postoperative pain and fever who are unable to take oral medications. Abdelbaser I et al. compared IV ibuprofen administered at 10 mg/kg every 6 hours with IV 0.5 mg/kg every 6 hours in 59 children aged between 2-8 years who underwent lower abdominal surgeries [6]. The authors concluded that postoperative opioid consumption, pain scores, and adverse events were comparable in both groups. However, the incidence of fever was significantly lower in the children who received IV ibuprofen (p=0.039). Haddadian A et al. randomized 150 patients with distal radius fractures to receive either 400 mg IV ibuprofen or 30 mg IV ketorolac infusion [36]. Upon analyzing the findings, the authors concluded that IV ketorolac 30 mg was more efficacious in alleviating pain in these patients than IV ibuprofen 400 mg. Shaker SH and Borghei SA compared the analgesic efficacy and safety of IV ibuprofen 800 mg with IV ketorolac 30 mg in 70 patients with renal colic (35 patients in each group) [2]. The authors concluded that although the analgesic efficacy of both NSAIDs was the same, they recommended ketorolac over ibuprofen due to the lower incidence of gastrointestinal effects with the former. Pinzon and Susanto compared the efficacy of IV ibuprofen 800 mg with IV ketorolac 30 mg in 60 patients with non-specific musculoskeletal pain [37]. The authors concluded that IV ibuprofen was more effective compared to IV ketorolac for improving sleep quality in patients with acute non-specific musculoskeletal pain. Forouzanfar MM et al. randomized 240 patients with acute renal colic to receive IV ibuprofen and IV ketorolac for pain relief [3]. The authors concluded that IV ibuprofen was much more rapid in onset for relieving acute colicky pain when compared to ketorolac. However, the adverse effect profiles of both drugs were comparable.

This review has several pertinent limitations. Although all studies included were RCTs, the types of surgeries were not identical, featuring different severities of postoperative pain. Several outcomes were not consistently reported by all the studies. Many outcomes essential for analysis were reported by only two studies. Studies did not explicitly report adverse events like bleeding, renal impairment, or gastrointestinal symptoms, which affected the statistical analysis. Thus, there was significant statistical and clinical heterogeneity in the included studies. The surgeries for which the medications were used were also not similar. Therefore, the results of this review must be interpreted with caution. Further, well-designed studies are needed to investigate the analgesic efficacy or non-inferiority of both NSAIDs.

## Conclusions

Based on the results from this review, both IV ketorolac and IV ibuprofen appear to be equally effective NSAIDs for managing acute postoperative pain. They showed comparable analgesic efficacy in terms of opioid requirements, pain scores, and patient satisfaction. However, the results of the present review should be interpreted with caution due to the clinical and statistical heterogeneity in the various studies, the high risk of bias involved, and the level of evidence for various outcomes. Further studies should explore various NSAIDs after addressing the limitations mentioned in this review.
